# Identification and Functional Characterization of the Transcription Factors AhR/ARNT in *Dendroctonus armandi*

**DOI:** 10.3390/cells11233856

**Published:** 2022-11-30

**Authors:** Bin Liu, Hui Chen

**Affiliations:** 1 State Key Laboratory for Conservation and Utilization of Subtropical Agro-Bioresources, Guangdong Laboratory for Lingnan Modern Agriculture, College of Forestry and Landscape Architecture, South China Agricultural University, Guangzhou 510642, China; 2 College of Forestry, Northwest A&F University, Xianyang 712100, China

**Keywords:** *Dendroctonus armandi*, AhR, ARNT, detoxification metabolism, transcriptional regulation

## Abstract

The aryl hydrocarbon receptor (AhR) and aryl hydrocarbon receptor nuclear translocator (ARNT) belong to the bHLH-PAS (basic Helix–Loop–Helix–Period/ARNT/Single-minded) family of transcription factors, which participate in the sensing and transmitting stimuli of exogenous and endogenous chemical substances, and subsequently activates genes transcription involved in various detoxification and physiological functions. However, they have not been identified in *Dendroctonus armandi,* and their roles in the detoxification metabolism are unclear. In the present study, *AhR* and *ARNT* of *D. armandi* were characterized. Spatiotemporal expression profiling indicated that *DaAhR* and *DaARNT* were highly expressed in the adult and larval stages of *D. armandi* and mainly expressed in the midgut and Malpighian tubules of adults. Additionally, the expression of *DaAhR* and *DaARNT* significantly increased after exposure to (−)-𝛽-pinene, (+)-3-carene, and (±)-limonene. Silencing *DaAhR* and *DaARNT* increased the susceptibility of *D. armandi* to (−)-𝛽-pinene, (+)-3-carene, and (±)-limonene, and the activities of detoxification enzyme were also remarkably reduced. Moreover, *DaCYP6DF1* and *DaGSTs2* were significantly down-regulated after injections of *dsAhR* and *dsARNT* in the male and female adults, with the expression of *DaCYP6DF1* decreasing by higher than 70%. The present study revealed that the transcription factors *AhR* and *ARNT* of *D. armandi* were induced by terpenoids and participated in the regulation of *DaCYP6DF*1 expression, which was associated with *D. armandi*’s susceptibility to (−)-𝛽-pinene and (±)-limonene. These results may provide a theoretical basis for the integrated control of *D. armandi* and improve our comprehension of insect toxicology.

## 1. Introduction

The Chinese white pine beetle, *Dendroctonus armandi* Tsai and Li (Coleoptera: Curculionidae: Scolytinae), is an aggressive and destructive pest in coniferous forests in the middle Qinling Mountains of China. It invades not only healthy *Pinus armandii* but also attracts other pests to the host trees, which has resulted in the destruction of the forest ecological system and caused serious economic losses [1]. An important period of the bark beetle’s life process is the host colonization stage, during which they must resist the host’s defenses to reproduce successfully [2]. The resistance of *P. armandii* to bark beetles mainly depends on the composition and induced physical and chemical defense, and the induced oleoresin terpenes are the main defense components [3]. Oleoresin is a complex composed of dozens of monoterpenes, diterpenes, and a few sesquiterpenes [4]. According to a previous study, 𝛼-pinene, 𝛽-pinene, limonene, myrcene, and camphene are the main compounds in the volatile oleoresin terpenes, which were derived from the resin of *P. armandi* [3]. Moreover, they can harm or kill beetles due to their toxic effects [5,6].

In insects, the detoxification of toxic chemicals mainly contains the enhancement of cytochrome P450 monooxygenase, glutathione *S*-transferase, and esterase activity levels [7]. The expression of detoxification genes directly affects the activity level of detoxification enzymes [8,9]. The increase of detoxification enzyme activity attributed to the upregulation of gene expression is likely to be influenced by transcriptional regulation, including *trans*-acting factors and *cis*-acting elements [10,11,12]. The former, also called transcription factors, bind to specific reaction elements on promoters and regulate the expression of target genes, and then activate or inhibit the transcription of related genes [13]. Three transcription factor superfamilies in insects are known as xenobiotic sensors that regulate the expression of detoxification genes, including basic leucine zipper (bZIP) proteins, nuclear receptors, and basic helix–loop–helix/Per–Arnt–Sim (bHLH-PAS) [14]. In *Plutella xylostella*, a transcription factor *FTZ-F1* affiliated with a nuclear receptor regulates the *CYP6BG1* expression, possibly improving its resistance to chlorantraniliprole [15]. In addition, as a bZIP transcription factor, *CncC* modulates the expression of several *GST* and *P450* genes to enhance malathion resistance in *Drosophila melanogaster* [16].

The family of bHLH-PAS proteins contains several dimeric transcription factors with multiple functions [17]. AHR is a member of the bHLH PAS protein family, which belongs to a ligand-activated transcription factor that regulates multiple xenobiotic responses [18]. In vertebrates, *AhR* contains *AhR1* and *AhR2*, with *AhR1* expressed in almost all vertebrates and *AhR2* only expressed in birds and fish. [19]. ARNT, as another bHLH-PAS protein family member, is a constitutive nuclear protein forming heterodimers with AhR [20]. Heterodimer recognizes and then combines with the xenobiotic response elements of target genes to mediate their expression [21]. The structure of transcription factor AhR/ARNT can be divided into bHLH, PAS, and transcriptional activation domains (TAD) from *N*- to *C* termini, the last of which primarily regulate the transcriptional activation of downstream related genes [22]. The *AhR* and *ARNT* have only one subtype in insects, which form heterodimers that participate in regulating the related detoxification gene expression [23,24,25]. For instance, *AhR*/*ARNT* regulates the *CYP6CY3* and *CYP6CY4* expression levels to enable *Myzus persicae* resistance to nicotine [23]. Similarly, the expression of *CYP6DA2* is also induced by *AhR*/*ARNT* in cotton aphids, which is involved in the resistance to spirotetramat [24]. Moreover, *AhR* is involved in chlorpyrifos susceptibility in *Locusta migratoria* by regulating the expression of *LmGSTd7* [25].

As we have never used pesticides against *D. armandi* in the sampling site, the host’s physical and chemical defenses are the main pressure on beetles. Transcription factors *AhR* and *ARNT* have not been characterized in *D. armandi,* and their roles in the metabolic process of detoxification are not fully clear. In the present study, we cloned *DaAhR* and *DaARNT* and then analyzed the gene structures. The effects of different terpenoids exposure on the expression level of *DaAhR* and *DaARNT* were performed with quantitative real-time PCR. Both genes were knocked down by RNA interference, and the *D. armandi* susceptibility to (−)-𝛼-pinene, (−)-𝛽-pinene, (+)-3-carene, and (±)-limonene was investigated. The expression of detoxification genes and their respective P450, GST, and CarE activity levels were also measured. Our study indicated the transcription factors *DaAhR* and *DaARNT* are involved in the xenobiotic metabolism of *D. armandi*. These results, which may provide a new perspective for these detoxification enzyme genes, are transcriptionally regulated and a theoretical basis for pest control.

## 2. Materials and Methods

### 2.1. Insects and Reagents Preparation

*D. armandi* were collected and reared as previously described [26]. (−)-𝛼-pinene (98%), (−)-𝛽-pinene (99%), (+)-3-carene (90%), (±)-limonene (95%), and Dimethyl sulfoxide (DMSO) were obtained from the Aladdin Industrial Corporation (Shanghai, China). 

### 2.2. RNA Extraction, cDNA Synthesis, and Reverse Transcription Quantitative PCR (qRT-qPCR) 

Total RNA was determined as previously described [27]. The relative expression level of each gene was determined by qRT-PCR. The PCR cycling conditions were performed as previously described [28]. The sequences of *β-actin* (accession number: KJ507199.1) and *CYP4G55* (accession number: JQ855658.1) in *D*. *armandi* were used as the reference genes [29,30]. The relative expression levels were analyzed by the 2^-ΔΔCt^ method [31]. All the primers are listed in Table S1.

### 2.3. Gene Cloning and Bioinformatic Analysis

The specific primers (shown in Table S1) were designed to clone the full-length cDNA sequences of *DaAhR* and *DaARNT.* The two obtained sequences were deposited in the GenBank, and their accession numbers are shown in Table 1. In addition, the open reading frames (ORFs) of cDNA sequences were obtained by ORF Finder (https://www.ncbi.nlm.nih.gov/orffinder/, accessed on 20 June 2022). Multiple sequence comparison of proteins was performed with DNAMAN 6.0. Molecular weight (kDa) and Isoelectric points were predicted by ProtParam (http://web.expasy.org/protparam/, accessed on 20 June 2022). MEGA 6.0 was used to construct the phylogenetic trees with the neighbor-joining method [32].

### 2.4. Developmental- and Tissue-Dependent Expression Profiles of DaAhR and DaARNT

*D. armandi* larvae were separated into the following stages: early larvae, late larvae, early pupae, late pupae, teneral adults, emerged adults, and feeding adults. The antennae, brain, hindgut, midgut, foregut, fat body, pheromone gland, hemolymph, and Malpighian tubules of emerged adults were collected by dissection and then stored at −80 °C. A total of three independent biological replicates were prepared for gene expression analysis.

### 2.5. Terpenoids Exposure

Fumigation treatment was performed as previously described [29]. The male and female of emerged adults were divided into six groups and treated with LC_50_ concentrations of (−)-𝛼-pinene, (−)-𝛽-pinene, (+)-3-carene, and (±)-limonene for 2 h in 20 mL glass vial [33]. The group of DMSO exposure was used as a control. Each treatment contained 40 adults of essentially the same size. After the adults regained their vitality, they were transferred to an artificial climate cabinet. To explore the effect of terpenoids on the expression of *DaAhR* and *DaANRT*, the surviving adults were collected at 24 h post-exposure to LC_50_ of each terpenoid. Meanwhile, the DMSO-treated surviving adults were collected as controls at the same time point. 

### 2.6. Double-Strand RNA (dsRNA) Synthesis and Injection

The synthesis of dsRNA was performed as previously described [26]. Briefly, the T7 Ribo-MAXTM Express RNAi System (Promega, Madison, MI, USA) was used for the synthesis of ds*GFP* (395 bp), ds*AhR* (412 bp), ds*ARNT* (387 bp), and ds*CYP6DF1* (455 bp). RNAi primers (Table S1) were designed based on the sequences obtained. Injection with ds*GFP* was used as a control. To prevent off-target effects, we chose specific target fragments to avoid any overlap with other genes, and the sequence specificity of target fragments was tested via NCBI BLAST. The final dsRNA products were diluted to 1000 ng/µL with diethylpyrocarbonate (DEPC)-treated water, then stored at –80 °C and used within 6 months. Before injection, *D. armandi* were placed in an ice bath for 10 min. The beetles were immobilized on an agarose plate using manual forceps. Afterward, each of the *D. armandi* emerged adults were microinjected with 0.2 µL dsRNA solution. Each treatment group contained 40 individuals, and 6 individuals from each treatment group were collected 24, 48, and 72 h after injection and then stored at –80 °C until qRT-PCR. Each treatment group contained three biological replicates. In addition, after injection at 48 h, beetles were also used for enzyme determination, and the P450, GST, and CarE activity levels were measured as previously described [7]. At the 48 h after dsRNA injection, the adults were treated with (−)-𝛼-pinene, (−)-𝛽-pinene, (+)-3-carene, and (±)-limonene as described previously, then they were reared in normal conditions for 48 h and the mortality rates were determined. We selected 12 genes from the three classes of *D. armandi* detoxifying enzymes: 6 CYP genes from the CYP3 clade, 4 GST genes from epsilon and sigma superfamilies, and 2 carboxylesterases, which are involved in the xenobiotic compounds’ detoxification in *D. armandi* [34,35]. These detoxification gene expression levels (*P450, CarE,* and *GST*) after dsRNA injection at 48 h were measured by qRT-PCR. The primers are listed in Table S1. Each treatment group contained three biological replicates.

### 2.7. Statistical Analysis

SPSS Statistics 19.0 (IBM, Chicago, IL, USA) was performed in all the statistical data analyses. Post-hoc Tukey tests were used to check the difference through one-way ANOVA. The two-sample analyses were performed with Student’s *t*-test. In addition, Prism 6.0 (GraphPad Software, San Diego, CA, USA) was used to plot graphs.

## 3. Results

### 3.1. Sequencing and Bioinformatic Analysis

**Figure 1:**
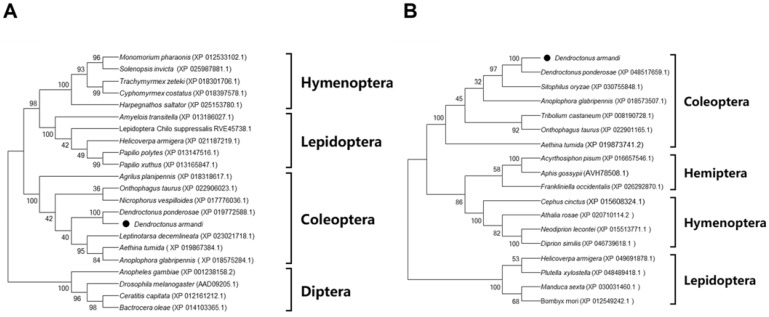
Phylogenetic analysis of *D. armandi* AhR (**A**) and ARNT (**B**) with other insect species. The phylogenetic tree was constructed in MEGA 6.0 using the neighbor-joining method. Bootstrap values (1000 replicates) are indicated next to the branches, and GenBank accession numbers are shown in parentheses. The black dot indicates *D. armandi* AhR and ARNT.

The full-length *D. armandi AhR* and *ARNT* cDNAs were cloned and characterized. The lengths of the coding regions of *AhR* and *ARNT* are 2412 bp and 2106 bp, which encode 803 and 701 amino acids, respectively. Moreover, the molecular weights (MW) of proteins of AhR and ARNT are 90.63 and 77.29 kDa, and the isoelectric points (PI) are 7.29 and 6.16, respectively (Table 1). A phylogenetic analysis showed that *DaAhR* (Figure 1A) and *DaARNT* (Figure 1B) have a high homology with their counterpart in *Dendroctonus ponderosa,* and they were also clustered with the Coleoptera group. An amino acid sequence alignment indicated that the bHLH and PAS domains of *DaAhR* and *DaARNT* are relatively conserved among different species (Figure S1).

### 3.2. Spatiotemporal Expression Pattern of DaAhR and DaARNT 

**Figure 2:**
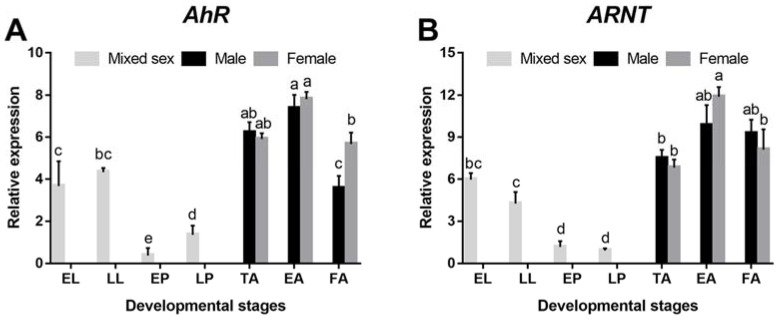
Relative expression levels of *AhR* (**A**) and *ARNT* (**B**) in different developmental stages of *D. armandi*. The relative expression levels were normalized with *β-actin* and *CYP4G55*. Different lowercase letters indicate significant differences at *p* < 0.05. Post-hoc Tukey tests following one-way analysis of variance (ANOVA). All values are mean ± *SE*, *n* = 3. EL, early larvae; LL, late larvae; EP, early pupae; LP, late pupae; TA, teneral adult; EA, emerged adult; FA, feeding adult.

**Figure 3:**
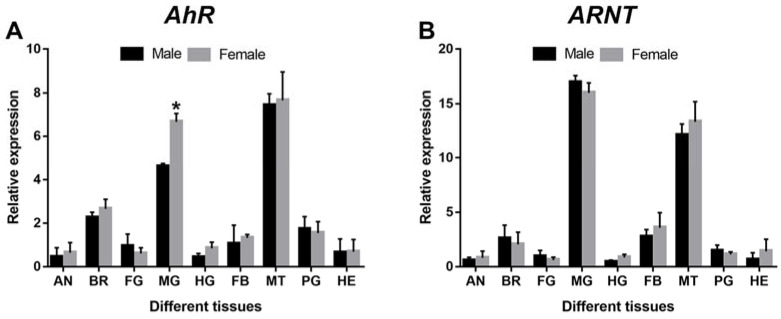
Relative expression levels of emerged adults of *AhR* (**A**) and *ARNT* (**B**) in different tissues of *D. armandi*. The relative expression levels were normalized with *β-actin* and *CYP4G55*. The asterisk indicates a significant difference between female and male expression levels (* *p* < 0.05, independent Student’s Test). All values are mean ± *SE*, *n* = 3. AN, antennae; BR, brain; FG, foregut; MG, midgut; HG, hindgut; FB, fat body; MT, Malpighian tubules; PG, pheromone gland; HE, hemolymph.

*AhR* and *ARNT* were expressed in all developmental stages of *D. armandi*. They were expressed the highest in the adult stage, followed by the larval stage, and the lowest in pupae (Figure 2). Moreover, as for the adult stage, the expression level of *AhR* in females was significantly higher than that in males in the feeding adults (Figure 2A). While *ARNT* showed the opposite result in the feeding adults, there was no statistical significance (Figure 2B). In addition, the expression of *AhR* and *ARNT* was found at different levels in tissues, and there were gender differences in some tissues. Specifically, *AhR* and *ARNT* were expressed predominantly in the midgut and Malpighian tubules (Figure 3). Moreover, the expression of *AhR* in female adults was higher than in male adults in the midgut (Figure 3).

### 3.3. Exposure to Terpenoids

**Figure 4:**
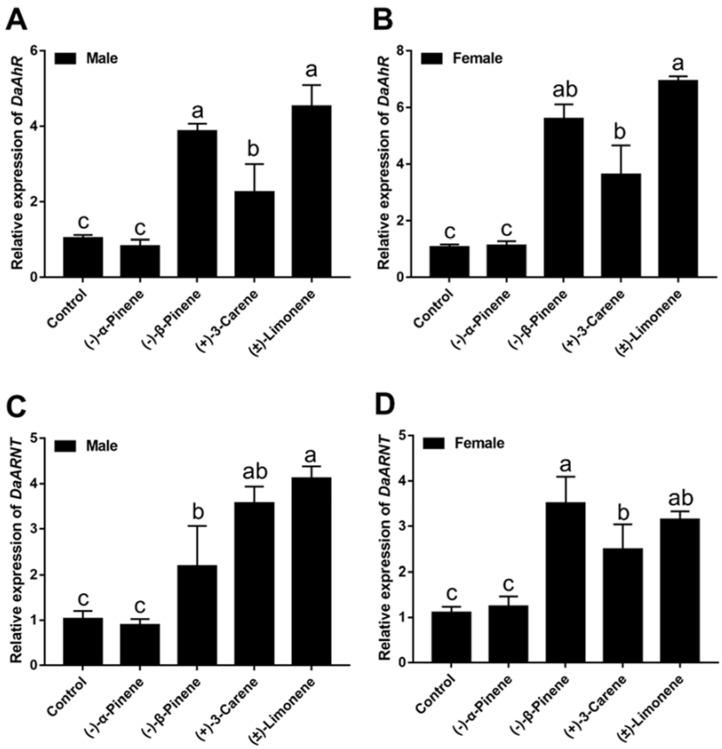
Relative expression levels of *AhR* and *ARNT* in male adults (**A**,**C**) and female adults (**B**,**D**) of *D. armandi* after stimulation with four terpenoids at an exposure time of 48 h. The relative expression levels were normalized with *β-actin* and *CYP4G55*. Different letters indicate significant differences at *p* < 0.05. Post-hoc Tukey tests following one-way analysis of variance (ANOVA). All values are mean ± *SE*, *n* = 3.

As shown in Figure 4 the expression of *DaAhR* and *DaARNT* can be induced to varying levels at 48 h after terpenoids treatment in male and female adults. After (−)-𝛼-pinene, (−)-𝛽-pinene, (+)-3-carene, and (±)-limonene exposure, the expression of *DaAhR* significantly increased by 2.72-, 1.17-, and 3.35-folds in male adults (Figure 4A), and 4.22-, 2.39-, and 5.46-folds in female adults (Figure 4B), respectively, as compared with DMSO-treated controls. *DaARNT* showed increases by 1.11-, 2.47-, and 2.99-folds in male adults (Figure 4C) and 2.17-, 1.25-, and 1.85-folds in female adults (Figure 4D), respectively. However, there was no significant change after (−)-𝛼-pinene treatment in *D. armandi.*

### 3.4. Functional Analysis of DaAhR and DaARNT by RNAi Silencing

**Figure 5:**
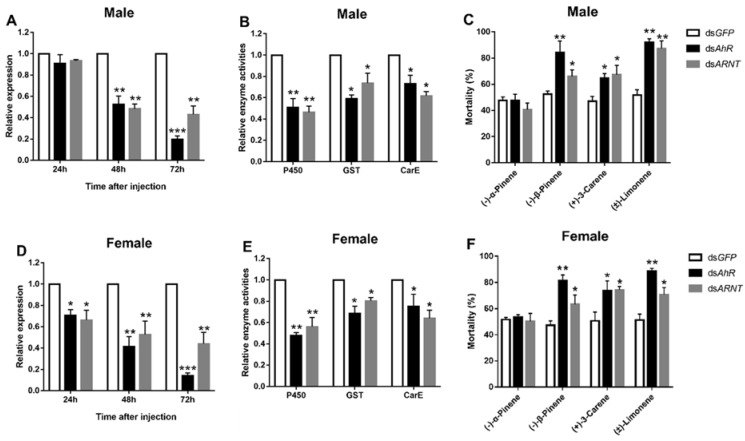
Functional analyses of *AhR* and *ARNT* by RNAi in *D. armandi* adults. (**A**,**D**) Relative expression levels of *DaAhR* and *DaARNT* in adults injected with dsRNA at 24, 48, and 72 h post-injection. (**B**,**E**) Relative detoxification enzyme activities after the silencing of *DaAhR* and *DaARNT*. (**C**,**F**) The mortality of adults exposed to four terpenoids was assessed at 48 h after dsRNA injection. The relative expression levels were normalized with *β-actin* and *CYP4G55*. The asterisk indicates a significant difference between treatment groups (* *p* < 0.05, ** *p <* 0.01, *** *p* < 0.001, independent Student’s Test). All values are mean ± *SE*, *n* = 3.

To further investigate the role of *DaAhR* and *DaARNT* in host chemical defense, the expression of two genes was repressed by RNAi in male and female adults. Compared with the control group, after dsRNA injection, the expression levels of *DaAhR* and *DaARNT* of adults were significantly downregulated at 24, 48, and 72 h, except for in male adults at 24 h (Figure 5A,D). In addition, the expression level of *DaAhR* and *DaARNT* decreased most at 72 h in male and female adults, reaching 80.3% and 57.0%, 86.0%, and 55.7%, respectively (Figure 5A,D). Moreover, the relative activities of P450, GST, and CarE remarkably reduced compared to the control after the injection of dsRNA (Figure 5B,E). Additionally, knocking down *DaAhR* and *DaARNT* increased the beetle’s susceptibility to terpenoids. The mortality of *dsAhR* and *dsARNT*-injected beetles elevated by 37.1% and 21.2%, 22.4% and 23.3, and 43.4% and 38.1% in male adults (Figure 5C), respectively, after the (−)-𝛽-pinene (+)-3-carene and (±)-limonene treatment when compared with the control. The female adults were enhanced by 34.3% and 18.7%, 20.3% and 20.1, and 36.4% and 19.5%, respectively (Figure 5F). Nevertheless, there was no significant change in (−)-𝛼-pinene susceptibility (Figure 5C,F). 

### 3.5. Analysis of DaAhR and DaARNT Regulation of Detoxification Genes

**Figure 6:**
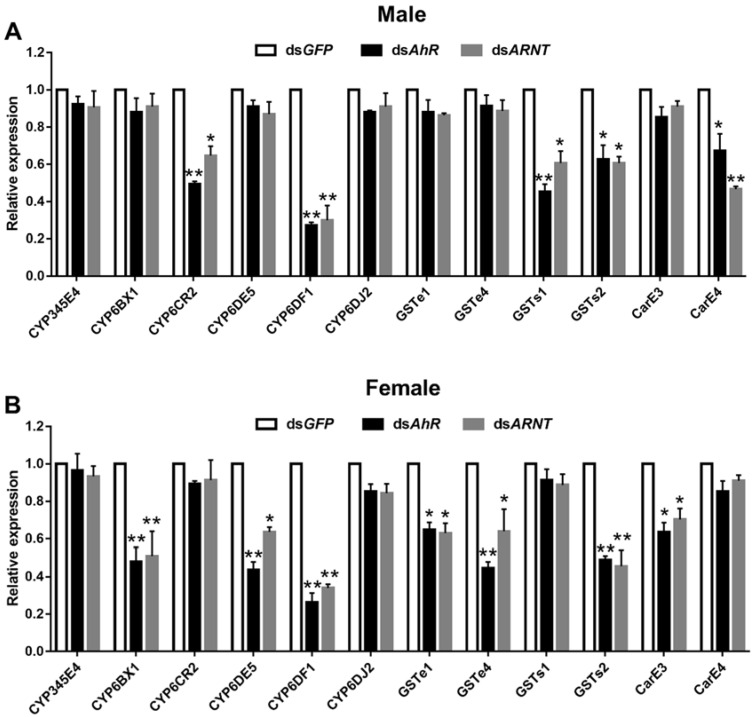
RT-qPCR analyses of the expression of 12 detoxification genes after silencing *AhR* (**A**) and *ARNT* (**B**) in *D. armandi* adults. The relative expression levels were normalized with *β-actin* and *CYP4G55*. The asterisk indicates a significant difference between treatment groups (* *p* < 0.05, ** *p <* 0.01, independent Student’s Test). All values are mean ± *SE*, *n* = 3.

To further explore the role of *DaAhR* and *DaARNT* in the detoxification metabolism of *D. armandi,* the expression levels of detoxification-related genes were determined using qRT-PCR when *DaAhR* and *DaARNT* were silenced. Among the *P450, GST,* and *CarE* genes, *CYP6CR2, CYP6DF1, GSTs1, GSTs2,* and *CarE4* expression levels were significantly downregulated when *DaAhR* and *DaARNT* were reduced in male adults (Figure 6A). The expression levels of *CYP6BX1, CYP6DE5, CYP6DF1, GSTe1, GSTe4, GSTs2,* and *CarE3* were significantly inhibited when silencing *DaAhR* and *DaARNT* in female adults (Figure 6B). These results indicated that *DaAhR* and *DaARNT* regulate the expression levels of detoxification genes and the metabolic detoxification enzyme activity of the corresponding genes participating in *D. armandi*’s susceptibility to host allelochemicals.

**Figure 7:**
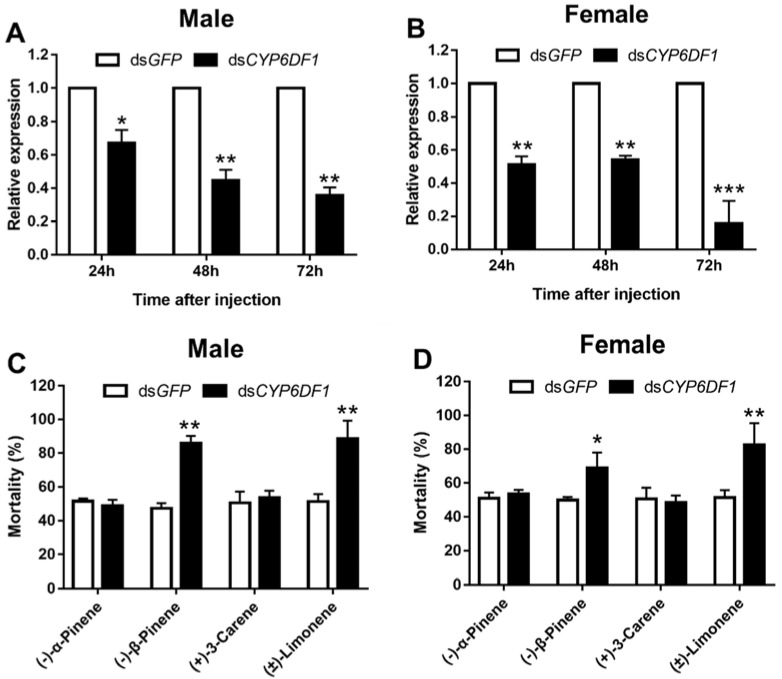
(**A**,**B**) Relative expression levels of *DaCYP6DF1* in adults injected with dsRNA at 24, 48, and 72 h post-injection. (**C**,**D**) The mortality of adults exposed to four terpenoids was assessed at 48 h after dsRNA injection. The relative expression levels were normalized with *β-actin* and *CYP4G55*. The asterisk indicates a significant difference between treatment groups (* *p* < 0.05, ** *p <* 0.01, *** *p* < 0.001, independent Student’s Test). All values are mean ± *SE*, *n* = 3.

Moreover, the expression of *CYP6DF1* and *GSTs2* significantly decreased after *DaAhR* and *DaARNT* injection, with *CYP6DF1* being reduced by higher than 70% (Figure 6). To further reveal the role of *CYP6DF1* in the detoxification metabolism, we determined the susceptibility of *D. armandi* to four terpenoids after silencing *CYP6DF1*. The expression of *DaCYP6DF1* in adults was down-regulated significantly at 24, 48, and 72 h when compared to the control after dsRNA injection. In addition, the expression level of *DaCYP6DF1* decreased the most at 72 h in male and female adults, reaching 81.7% and 62.4%, respectively (Figure 7A,B). The mortality significantly elevated in the *dsCYP6DF1*-injected group after treatment with (−)-𝛽-pinene and (±)-limonene compared with the control, with 38.7% and 37.3% in male adults, 21.4% and 31.3% in female adults, respectively (Figure 7C,D). Nevertheless, there was no significant change in (−)-𝛼-pinene and (+)-3-carene susceptibility.

## 4. Discussion

During *D. armandi* attack host trees, they must overcome the induced defense system of the pine trees, which contains a large number of terpenoids [3]. To avoid host toxicity, insects have evolved a complicated regulatory mechanism to solve xenobiotics exposure, including enhanced detoxification metabolisms [36]. Although the relationship between the induction of relevant detoxification genes and insect tolerance to exogenous chemicals has been widely reported, the regulatory cascades of these xenobiotic response genes in *D. armandi* are still basically unknown. In the present study, we clarified the effects of transcription factors DaAhR and DaARNT on *D. armandi* downstream-related detoxification genes and relevant regulation metabolism in plant toxin resistance.

Stage-dependent expression patterns of *DaAhR* and *DaARNT* were detected in different developmental stages with RT-qPCR, and the results indicated that *DaAhR* and *DaARNT* were expressed in all developmental stages of *D. armandi*. The highest expression level was detected in the larval and adult stage, and the lowest was in the pupae, which may be involved in detoxifying xenobiotics in food. Moreover, the expression of *DaAhR* and *DaARNT* was further investigated in various tissues of adults, suggesting that it was expressed predominantly in the midgut and Malpighian tubules compared to other parts. The midgut is not only the place where insects digest and absorb but also plays a key role in resisting exogenous substances [37]. Previous studies have reported that the Malpighian tube also has a function in xenobiotics detoxification [38,39]. It was worth noting that the expression of *DaAhR* in female adults was higher than in male adults in the feeding adults and midgut, while there was no gender difference in *DaARNT*. This indicates that *DaAhR* plays an important role in the location and detoxification of host volatiles in female adults. Therefore, the overexpression of *DaAhR* and *DaARNT* in the detoxification organ of *D. armandi* may play a key role in the detoxification of exogenous substances. Further studies can be performed to show the function of *DaAhR* and *DaARNT* in other different developmental stages and tissues of insects.

Under normal circumstances, the increased metabolism in insects arises from the increased detoxification effects attributed to amplification, constitutive overexpression, and mutation of genes [40]. Elements in promoters and transcription factors mediate transcriptional regulation and participate in the expression of detoxification genes. The suppression of hepatocyte nuclear factor 4 by transcription factor results in the resistance to imidacloprid in *N. lugens* through regulating UDP-glycosyltransferase (UGT) and P450 genes [41]. The promoter region motif may be a candidate region for the transcription of tolerance-related genes [42,43]. A *cis*-acting element was called a xenobiotic response element (XRE) of flavonoids in *Helicoverpa zea*, which mediated the expression of *CYP321A1* induced by flavonoids [44]. In *Spodoptera litura, Nrf2* activated the *GSTe1* expression by binding to the *cis*-acting element in its promoter in response to insecticides and phytochemicals [45]. As vital transcription factors for xenobiotic sensors, AhR and ARNT regulate a large number of metabolic genes in various insect species [10]; however, their roles in *D. armandi* detoxification remain unclear. 

In the present study, the expression of *DaAhR* and *DaARNT* significantly increased after treatment with (−)-𝛽-pinene, (+)-3-carene, and (±)-limonene, and silencing *DaAhR* and *DaARNT* enhanced the susceptibility of *D. armandi*, with the decrease of enzyme activity level corresponding to greater sensitivity. This revealed that the AhR/ ARNT signal pathway in *D. armandi* may be involved in metabolic responses to environmental stresses associated with host allelochemical exposure. In addition, *DaAhR* and *DaARNT* are homologous to other species. For instance, the AhR signal pathway is induced when mediating chemical-induced developmental toxicity in zebrafish [46]. Silencing *LmAhR* enhanced the susceptibility to chlorpyrifos in *L. migratoria* [25]. Moreover, the expression of the transcription factor *SlituCncC* is induced after indoxacarb exposure in *S. litura* [47]. Additionally, silencing *NlAhR* and *NlARNT* affects isoprocarb, imidacloprid, and etofenprox susceptibility in *N. lugens* [48]. 

The change in detoxification enzyme activity is due to the change in the expression of detoxification genes. In this study, silencing *DaAhR* and *DaARNT* resulted in the differential downregulation of some detoxification enzyme genes, with *DaCYP6DF1* being downregulated by higher than 70%. These results indicated that *DaCYP6DF1* was supposed to be a candidate gene that mainly participated in host chemical susceptibility. Similar results have also been reported in other insects. For example, AhR/ARNT of *M. persicae* regulated the expression of *CYP6CY3* and *CYP6CY4* cooperatively, leading to the nicotine adaption to tobacco [23]. The *cis*-regulatory elements *AhR*/*ARNT* and *CncC*/*Maf* participated in the expression regulation of some *GST* detoxification enzyme genes in *Spodoptera exigua*. Moreover, the constitutive overexpression of *CncC, Maf,* and *AhR* promoted the increased expression of several detoxification-related genes and led to chlorpyrifos and cypermethrin resistance [49]. In addition, *NlAhR* and *NlARNT* were induced by pesticides and participated in the *NlCarE7* regulation, which was involved in the susceptibility to etofenprox and isoprocarb in *N. lugens* [48]. Previous studies have reported that *CYP* genes in *D. armandi* were remarkably increased in response to (−)-𝛼-pinene, (−)-𝛽-pinene, (+)-3-carene, and (±)-limonene treatments [29,34,35]. Because knockdowns of *DaCYP6DF*1 significantly increased *D. armandi* susceptibility to (−)-𝛽-pinene and (±)-limonene, we supposed that *DaAhR* and *DaARNT* regulate the susceptibility by targeting *DaCYP6DF*1.

## 5. Conclusions 

These results indicated that the transcription factors *DaAhR* and *DaARNT* of *D. armandi* were induced by terpenoids and involved in mediating the expression level of *DaCYP6DF*1, which was associated with *D. armandi* susceptibility to (−)-𝛽-pinene and (±)-limonene. Our study showed the potential roles of *DaAhR* and *DaARNT* in the detoxification metabolism of *D. armandi*. These findings uncovered the molecular mechanisms of host allelochemical detoxification in insects and may provide a theoretical basis for controlling this pest.

## Figures and Tables

**Table 1:** Physicochemical properties of putative *D. armandi* AhR and ARNT proteins.

Gene Name	Accession No	ORF (bp) ^a^	Amino Acid Residues ^a^	MW (KDa) ^a^	IP ^a^
*AhR*	OP378567	2412	803	90.63	7.29
*ARNT*	ON378568	2106	701	77.29	6.16

Note: ^a^ As predicted by BLAST (http://www.ncbi.nlm.nih.gov, accessed on 20 June 2022).

## Data Availability

All data are included in the text and Supplementary Materials.

## Supplementary Materials

The following supporting information can be downloaded at: https://www.mdpi.com/article/10.3390/cells11233856/s1, Figure S1: Multiple amino acid sequence alignments of DaAhR (A) and DaARNT (B) with six other insect species. Three characteristic domains (bHLH, PAS-A and PAS-B) of DaAhR and DaARNT are boxed in red. Table S1: Primer sequences used in the research.
