# Supplementary material for: Identification and Functional Characterization of the Transcription Factors AhR/ARNT in Dendroctonus armandi
Source: Cells. 2022 Nov 30;11(23):3856. doi: 10.3390/cells11233856 (PMC9736963; doi:10.3390/cells11233856)
Supplement: Supplementary file 1 [file cells-11-03856-s001.zip › cells-1962581-supplementary.pdf]

*Supplementary Material*

**Table S1.** Primer sequences used in the research

| Gene | Sequence （5′ → 3′） | | purpose |
|---|---|---|---|
| | **Forward** | **Reverse** | |
| *AhR* | ATGAACCAGTTGGGTACTGT | TCACAATACCATCACCAGTC | Full-length |
| | CTGTACGGTTTCGGTGTTTG | TCACTTCTTTTTGGGGGATT | qPCR |
| | **taatacgactcactatagggg**CTGAAGACCGGGAACAACTT | **taatacgactcactatagggg**CTGAATACCCCAGCAACAAT | RNAi |
| *ARNT* | ATGTACGCGTATGCCGGTGG | CTATTCAAAGGTGCCATCGA | Full-length |
| | GCCCCCACCGACTTATCTTC | CGTCCTCCATCCTTGCGTAT | qPCR |
| | ATCCTGCCTTTACGATAACA | TCACTTTGCCTCTCCATTTG | RNAi |
| *CYP345E4* | **TTATTGAAGAAAGCCAGGACA** | CACGGACGCAAAGTTCATACA | qPCR |
| *CYP6BX1* | TGACGGCATTCTTTCTTAT | CATCACTTCCTGGTACTACAT | qPCR |
| *CYP6CR2* | AACCCTTAACAAGCACAGAG | GATCATACCCAACAATTCCA | qPCR |
| *CYP6DE5* | ACTGCGGAACGAAATTATTG | GTCTTTGGTACATCGCCTGG | qPCR |
| *CYP6DF1* | TACTTCCCCGACCCCCATAA | CTGAGCAACGCAGCCAATCC | qPCR |
| | TTGGGAAAGAAATGGGGTGA | TGCGGCTGGATGTGAAGGTG | RNAi |
| *CYP6DJ2* | GTTAGTTTGCTTTTGTGCGT | CGATTTTGTATCCTTTCTTT | qPCR |
| *GSTe1* | TTTGGGCTTGGATGTAGAAT | TTGAAGTGTTGGAACCGTGT | qPCR |
| *GSTe4* | TGATGACGGCTCGAATA | GCATGACTGTCCCAAAT | qPCR |
| *GSTs1* | TAATGAAGGAACCAGACCCAA | GTCCAATAGCACGGCAAAGA | qPCR |
| *GSTs2* | GGAACTACTCCTGCTGGTCAAC | CGTATCAACGGCGGCATCAAT | qPCR |
| *CarE3* | AGATGCAGCAGAGGACAGTCACATG | GCTCCATTACGAAGACCGCCACCAT | qPCR |
| *CarE4* | GGCAATATTCGTATGCAGCGGCGTT | CCAGGTCGTTCGGCTTCGTGAGTT | qPCR |
| *GFP* | **taatacgactcactatagggg****ATGGTGTTCAATGCTTTTCA** | **taatacgactcactatagggg**CTCTCTTTTCGTTGGGGTCT | RNAi |

Note: T7 promoter sequences in the dsRNA synthesis primers were indicated in lowercase letters.

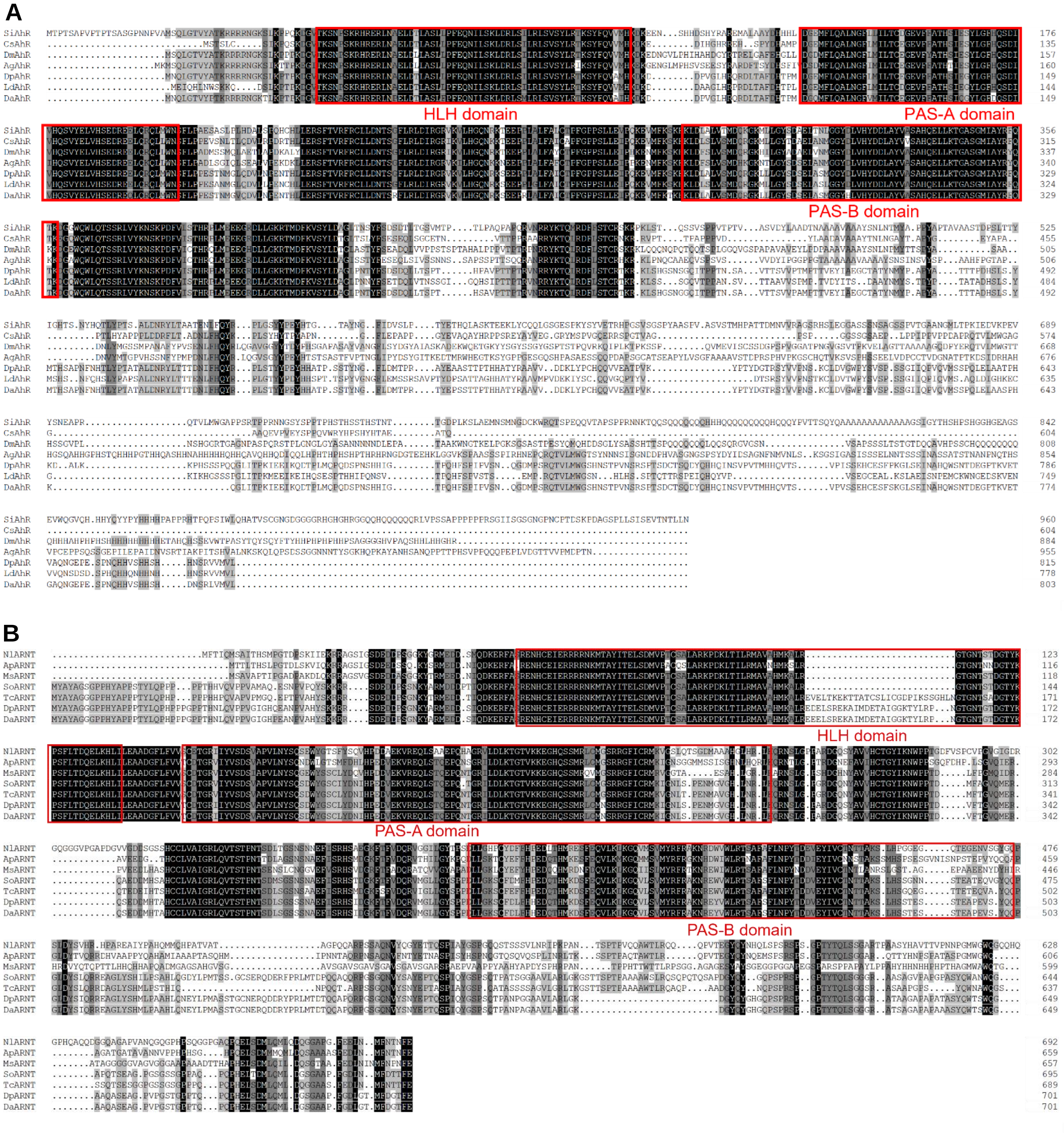


**Figure. S1.** Multiple amino acid sequence alignments of *DaAhR* (A) and *DaARNT* (B) with six other insect species. Three characteristic domains (bHLH, PAS-A and PAS-B) of *DaAhR* and *DaARNT* are boxed in red.
